# Final-year medical students need to know their future supervisory role of clinical associates

**DOI:** 10.4102/safp.v62i1.5019

**Published:** 2020-07-16

**Authors:** Michiel Koortzen, Lourens W. Biggs, Jacqueline Wolvaardt, Astrid Turner, Martin Bac, Mark Volpe

**Affiliations:** 1 School of Medicine, University of Pretoria, Pretoria, South Africa; 2 School of Health Systems and Public Health, University of Pretoria, Pretoria, South Africa; 3 Department of Family Medicine, School of Medicine, University of Pretoria, Pretoria, South Africa; 4 University of Saint Joseph, West Hartford, Connecticut, United States

**Keywords:** Clinical associate, bachelor of clinical medical practice, clinical supervision, doctors, medical students, ClinA

## Abstract

**Background:**

A clinical associate (ClinA) is a mid-level health professional who may only practise under the supervision of a medical doctor. By extension, medical students need to be prepared for this responsibility. This study explored whether final-year medical students at one university were aware of this supervisory role, felt prepared and were knowledgeable about the ClinAs’ scope of practice.

**Methods:**

A descriptive, cross-sectional study was conducted. The population included all final-year medical students who had completed their District Health and Community Obstetrics rotations (March to November 2017). After an end-of-rotation session, 151 students were given questionnaires to complete. A list of 20 treatments or procedures was extracted from the ClinAs’ gazetted scope of practice for a ‘knowledge test’. Data were analysed with Stata and Microsoft Excel. Ethical permission was granted.

**Results:**

The response rate was 77.4% (*n*/*N* = 117/151). The majority of participants (76.1%, *n* = 86) had worked with a qualified or student ClinA before and had a generally positive impression (81.4%; *n* = 70). Almost half (47.8%; *n* = 56) thought that the ClinAs’ scope of work was similar to registered nurses rather than a doctor’s (38.2%; *n* = 44). Most were unaware that they would be required to supervise ClinAs once qualified (65.8%; *n* = 77). On average, participants identified 12 out of 20 treatments or procedures that a ClinA could perform.

**Conclusion:**

Despite having worked with ClinAs, participants appeared largely unaware of their future legal obligation of supervision. Adequate clinical supervision is based on the knowledge of the scope of practice, which was variable. Formal training on the scope of the work of ClinAs is needed to prepare future doctors for their supervisory role. Medical schools have an obligation to adequately prepare their students in this regard as part of their transformative education with elements of interprofessional education.

## Introduction

For the first time in South African history, medical doctors are responsible for the clinical work of another cadre of health professional. This development has arisen from the fact that South Africa has a complex, multiple burden of disease and a concurrent shortage of health professionals – in particular medical doctors – in the public sector. One of the government’s responses to this shortage was to create a new category of health professional: the clinical associate (ClinA). Clinical associates are mid-level health professionals primarily intended to be employed in understaffed facilities in the public sector, thereby enabling government to achieve universal healthcare and allowing for the implementation of the National Health Insurance (NHI).^1^

Clinical associates complete a Bachelor of Clinical Medical Practice (BCMP) degree, which is a three-year degree and is currently offered by three universities in South Africa: the Walter Sisulu University, the University of the Witwatersrand and the University of Pretoria (UP). The BCMP students spend the first semester primarily attending classes at a medical school, with regular patient contact. These students continue their training at district hospitals and primary health-care clinics for the remainder of their studies.^2^ Teaching at these sites integrates theory and practice and family physicians are chiefly responsible for this teaching. Being taught at medical schools by family physicians is intended to foster a close and interdependent relationship between the two cadres of ClinAs and medical doctors.^3^ The education strategy is problem-based, patient-centred and self-directed with an emphasis on self-learning.^4,5,6^

According to communication with the Health Professions Council of South Africa, the training of ClinAs has accelerated from the first 2008 intake of the programme, with 851 trained ClinAs and 509 BCMP students registered in 2019. The first graduates entered the job market in 2011 and were favourably received in their respective district hospitals.^3^

The ClinAs’ scope of work is designed to avoid overlap with primary care nurse practitioners and includes consultations, physical examinations and counselling.^7^ In addition, they can assist with emergency care, and routine diagnostic and therapeutic procedures (including minor surgical procedures) as well as in-patient care. One of the design features of the ClinA programme includes adequate supervision and support by doctors to strengthen the quality of care provided by this cadre.^8^ A 2013 assessment of ClinAs’ experiences revealed not only challenges with doctors’ capacity for this supervisory role but also potential tensions between health professionals regarding the scope of practice.^8^

The perceptions of district managers and other key informants about the training and curricula of mid-level workers in four African countries, including ClinAs in South Africa, was explored recently.^9^ It found that the critical role mid-level workers play in health systems could be optimised even further with revised curricula and training in terms of content and pedagogical methods and could include interprofessional education. However, the authors do state that as it was a cross-sectional, rapid appraisal, they were unable to conduct a comprehensive evaluation of training at institutions.^9^

Once qualified, ClinAs may only legally practise under the supervision of a medical doctor. At UP there are no formalised opportunities in the curriculum for medical students to work in teams with ClinAs and become familiar with their scope of practice. Therefore, as a first step, a research study was conducted among the final-year medical students at UP to explore if they are aware of their future legal obligation and whether they feel prepared for this role.

## Methods

A descriptive, cross-sectional study was conducted at UP. The population included all final-year medical students who completed their District Health and Community Obstetrics rotations between March and November 2017.

After an end-of-rotation reflection session at the Department of Family Medicine, 151 students were given hard copy questionnaires to complete. Participation was voluntary and participants had the opportunity to decline from participating in the study or to withdraw at any stage. The questionnaire was adapted, with permission, from previous work done by Volpe et al. on physician assistants (PAs), the American equivalent of ClinAs.^10^ The questionnaire had seven closed-ended questions, one open-ended question, and a ‘knowledge test’ consisting of 20 treatments or procedures that were extracted from the ClinAs’ gazetted scope of practice.^7^ The questionnaire was piloted with the first group of final-year medical students and minor amendments were made to the questionnaire. Questionnaires were available in English only. Data were analysed with Stata/IC 14.2 (2018) and Microsoft Excel (2016).

### Ethical consideration

Ethical permission was granted by the Faculty of Health Sciences Research Ethics Committee (#53/2017).

## Results

The response rate was 77.4% (*n*/*N* = 117/151). A small proportion of the participants had a previous degree (15.4%; *n* = 18), none of which was a BCMP degree. The majority of participants (76.1%; *n* = 86) had worked with a ClinA (or a BCMP student) and characterised the experience as positive (81.4%; *n* = 70). The largest proportion of participants thought the work of ClinAs to be most similar to registered nurses (47.8%; *n* = 56) rather than medical doctors (38.2%; *n* = 44).

Very few participants (12.8%; *n* = 15) knew that they would be legally liable for the actions of ClinAs in the future, while approximately one-fifth were unsure (21.4%; *n* = 25). Among the 15 participants who said they knew about this future supervisory role, only seven students (46.7%) felt that they had received adequate training to fulfil this role and were comfortable with the responsibility.

In the ‘knowledge test’ about the ClinAs’ scope of work, on average the participants correctly identified 12 out of the 20 treatments or procedures as tasks a ClinA may perform (Table 1).

**TABLE 1:** Comparison of the responses to treatments or procedures presented as ‘A clinical associate can legally perform the following in accordance with their scope of work’.

Number	Treatments or procedures	Yes		No		Unsure	
		Frequency	%	Frequency	%	Frequency	%
1	Arterial blood gas (*N* = 116)	97	83.6	8	6.9	11	9.5
2	Assist in closed fracture reduction (*N* = 116)	90	77.6	17	14.7	9	7.8
3	Assist with caesarean sections	60	51.3	39	33.3	18	15.4
4	Assist in neonatal lumbar puncture (*N* = 116)	37	31.9	61	52.6	18	15.5
5	Assist in neonatal resuscitation (*N* = 115)	103	89.6	8	7.0	4	3.5
6	Assist in normal vaginal delivery (*N* = 116)	111	95.7	3	2.6	2	1.7
7	Bone marrow aspiration (*N* = 116)	23	19.8	65	56.0	28	24.1
8	Cricothyroidotomy (*N* = 116)	29	25.0	61	52.6	26	22.4
9	Full spine immobilisation and log roll (*N* = 116)	105	90.5	9	7.8	2	1.7
10	Insert an intrauterine contraceptive device (*N* = 116)	80	69.0	24	20.7	12	0.3
11	Mental health exam (*N* = 116)	91	78.4	18	15.5	7	6.0
12	Oral endotracheal intubation (*N* = 115)	53	46.1	39	33.9	23	20.0
13	Order and interpret blood tests (*N* = 116)	60	51.7	45	38.8	11	9.5
14	Order and interpret X-rays (*N* = 116)	61	52.6	43	37.1	12	10.3
15	Pap smear (*N* = 117)	107	91.5	7	6.0	3	2.6
16	Pleural biopsy (*N* = 114)	24	21.1	69	60.5	21	18.4
17	Set up an IV infusion (*N* = 117)	115	98.3	0	0	2	1.7
18	Share bad news or debrief a patient or a patient’s family (*N* = 117)	81	69.2	25	21.4	11	9.4
19	Sign a sick leave certificate (*N* = 117)	39	33.3	61	52.1	17	14.5
20	Suturing (*N* = 114)	104	91.2	7	6.1	3	2.6

## Discussion

This study explored whether final-year medical students at UP were aware of their future supervisory role, felt prepared for this role and were aware of the ClinAs’ scope of practice in order to be able to supervise them. The majority of participants were currently completing their first degree and had previously worked with a ClinA or a BCMP student. Those who had done so characterised the interaction as positive.

The most common (although less than half) comparison of a ClinA’s work was to that of a professional nurse – an unexpected response from medical students who train at the same institution. Although both the scopes of practice of ClinAs and of medical doctors are quite clear, there is a lack of both local and global literature regarding the perceptions and expectations that medical students have about ClinAs. In the few studies conducted, researchers noted that senior medical students had accurate expectations and good perceptions of PAs.^10^

That only seven participants were aware of their future supervisory role with regard to ClinAs is not surprising as, despite training at the same institution, medical students are not formally presented with the ClinAs’ role in the health-care system, their scope of practice or their requirement to be supervised by a medical doctor. Role clarification with constructive supervision is important for an effective, multidisciplinary team approach in health as well as quality improvement.^11,12^ By understanding the scope of practice of ClinAs and ensuring that their supervision is appropriate, the likelihood of healthcare team members clashing over the exact nature of their respective roles is mitigated.^3^ Even a small intervention such as reading a one-page document that described the education and role of PAs in healthcare in an American study showed that the understanding of the role of PAs was statistically significantly improved (*p* < 0.001) in the surveyed population thereafter.^10^

In this study, participants were mostly able to correctly identify the procedures that ClinAs were allowed to perform (based on the guidelines at the time). One possible explanation for this positive finding is that the examples given were similar to what students might see nurses do and which are also minimally to moderately invasive procedures. The common incorrect answers were either interventions (e.g. assist with caesarean sections) or medico-legal actions (e.g. sign a sick leave certificate) that participants might more readily associate with doctors. This finding is similar to what was reported in the American study, where the tasks that were more complex or required more knowledge were less likely to be identified as ones that a PA could perform.^10^

In a chapter of the *2019 South African Health Review*, the authors argue that the specific wording in the scope of practice does not enable the appropriate supervision needed for ClinAs and other team members to be effective. They advocate for an environment that ‘…. should allow for an optimal balance between direct and indirect supervision, in which the risks are managed dependent on the complexity of the task(s) and the competence of the clinical associate.’^13^

The example they provide is the presence of a ClinA in teams with doctors during the years of internship and/or community service.^13^ However, there is an opportunity during the undergraduate years of both professions to begin planting the seeds for this mutually supportive, collaborative environment.

This strategy would align with the call for more mechanisms of interprofessional education (‘when students from two or more professions learn about, from and with each other to enable effective collaboration and improve health outcomes’) and collaborative practice (‘when multiple health workers from different professional backgrounds work together with patients, families, carers and communities to deliver the highest quality of care’), which was the focus of the 2010 World Health Organization’s (WHO’s) Framework for Action on Interprofessional Education and Collaborative Practice.^14^ The benefits of an applied model that combines different pedagogies enables medical students to appreciate the role of other cadres in the modern-day multi-disciplinary environment.^15^

Being taught at medical schools is intended to foster a close and interdependent relationship between the two cadres,^3^ but based on the gaps in knowledge in this study, it could be argued that this interdependent relationship has not yet been extended to the medical students in the same training institution on a regular basis. This gap has implications for their future relationship where South African doctors remain ultimately responsible for the care that is provided and as such need to know the scope of practice of ClinAs to ensure optimal and safe care. Finally, if ClinAs are intended to serve in underserved and rural populations, it is critical for the doctors in these areas to know both what is included and excluded in the ClinAs’ scope of practice.

## Study limitations

The final response rate of 77.4% was affected by the inconvenient timing of the administration of the questionnaire as students wanted to leave after the academic session. The external validity of the study findings to other institutions or medical students was also reduced as this study was only conducted at one institution and did not include a comparison group with medical students from a university that does not train ClinAs. The lack of literature about the awareness of medical students about other cadres such as nursing professionals meant that information could not be validated. Finally, the ClinAs’ scope of practice has since been amended and the item ‘assist with neonatal lumbar puncture’ is no longer included.

## Conclusion

As the number of ClinAs continues to rise, there is a need for a ‘collaborative practice-ready’ health workforce to operate in a complex health environment. Therefore, it is imperative that medical students are aware of their future supervisory role, are adequately prepared for this role and are fully aware of the ClinAs’ scope of practice. Medical schools have an obligation to adequately prepare their students in this regard as part of their transformative education with elements of interprofessional education.^16^ Where appropriate and needed, the three universities that train both medical students and ClinAs can consider interprofessional learning opportunities not only for these two cadres but between others as well, while the other universities will have to explore solutions. The consequences of not addressing this role preparation among the medical students at UP could include ClinAs being underutilised or not being adequately supervised in the settings in which they practise, which will ultimately have an impact on quality of care.
